# Molecular Dynamics Simulation of Nano-Defects on Fused Silica Surface Induced by Low-Temperature Plasma Cleaning

**DOI:** 10.3390/molecules30163418

**Published:** 2025-08-19

**Authors:** Yuhai Li, Yilan Jiang, Laixi Sun, Qiang Yuan, Peng Zhang, Qingshun Bai, Xiaodong Yuan

**Affiliations:** 1 Laser Fusion Research Center, China Academy of Engineering Physics, Mianyang 621900, China; 15546027805@163.com (Y.L.); jiangyilan1023@163.com (Y.J.); sunlaixi@126.com (L.S.); 2 School of Mechatronics Engineering, Harbin Institute of Technology, Harbin 150000, China; zp@hit.edu.cn (P.Z.); qshbai@hit.edu.cn (Q.B.)

**Keywords:** fused silica, plasma cleaning, damage evolution, nano-defects, molecular dynamics

## Abstract

Low-temperature plasma cleaning technology has been widely used to clean various optical components precisely. After the complete removal of organic contaminants from fused silica surfaces through plasma cleaning, continuous plasma irradiation can lead to nano-defects on the fused silica surface, resulting in the degradation of optical performance. Thus, the microscale processes underlying plasma-induced surface damage on fused silica were investigated through molecular dynamics simulations, aiming to analyze the mechanisms of surface damage on optical components during plasma cleaning. Oxygen plasma bombardment disrupted fused silica bonds, leading to the successive sputtering of silicon–oxygen atoms. The quantity of sputtered silicon atoms demonstrated a linear correlation with irradiation time. The emergence of pit defects and distinctive interface damage patterns elucidated the impact of neutral oxygen atoms. Critical findings underscore the onset of significant damage beyond 33 eV, underlining plasma’s role in thinning fused silica. Temperature is a crucial factor affecting surface damage during plasma cleaning. Ultimately, investigating the surface damage mechanism of fused silica during plasma cleaning establishes a groundwork for achieving non-destructive optics cleaning.

## 1. Introduction

As the performance metrics of large precision optical instruments continue to advance, the cleanliness requirements for the surfaces of optical components are becoming increasingly stringent [1,2,3,4,5,6,7]. To ensure the initial cleanliness of the system, precision optical instruments have established a closed-loop system of ultra-clean manufacturing, assembly, maintenance, and inspection technologies [8,9,10,11]. Nevertheless, even after prolonged use, the optical performance of optical components continues to degrade gradually, primarily due to organic contamination and damage on the surfaces of optical components within vacuum environments [12,13,14,15]. In situ removal methods for organic contaminants on optical component surfaces have been progressively developed, introducing innovative dry cleaning techniques such as plasma cleaning [4,5,6,7], laser cleaning [16], and ultraviolet-ozone cleaning [17]. Among these, plasma cleaning technology, as an advanced optical post-processing method, can achieve in situ, efficient, and controlled removal of organic contaminants, addressing the shortcomings of traditional in situ cleaning techniques. Thus, low-temperature plasma cleaning technology has gradually become a mainstream method for in situ cleanliness of optical component surfaces in vacuum environments.

The electron temperature of radio frequency plasma is approximately ~3 eV, while the sheath voltage can reach several hundred volts [18]. Charged ions passing through the sheath are accelerated and bombarded onto the material surface, transferring energy to the lattice atoms on the material surface, inducing instantaneous dissociation reactions (10^−11^–10^−12^ s), which to some extent promote surface chemical reactions but also cause irreversible damage to the surface [3,19]. As the plasma cleaning process progresses, organic contaminants adsorbed on the surface of optical components gradually undergo molecular dissociation and desorption [20]. However, once surface organic contaminants are entirely removed, continuous plasma irradiation, or over-cleaning, can lead to lattice damage on the material surface, resulting in the formation of new defects [3,19]. The fundamental difference between plasma cleaning and processes such as film deposition, etching, and ion beam implantation lies in the disparity in particle kinetic energy and flux within the ionized gas. Thus, the core issue in plasma cleaning of optics surfaces is that after the removal of surface organic contaminants, the exposed optical component surface is subjected to continuous bombardment by reactive species (ions, free radicals, electrons, etc.) in the plasma, leading to alterations in its surface physical structure, an increase in roughness values, and sputtering of the surrounding vacuum chamber walls.

During the plasma cleaning process, it is essential to control the discharge parameters and effectively manage the cleaning to prevent damage to the surfaces of optical components. Maffini et al. [21] conducted in situ plasma cleaning of metal reflectors in 2017 to address material surface damage during cleaning. After 21 h of plasma cleaning of reflectors contaminated with metal particles, while some surface contaminants were effectively removed, the reflectance did not fully recover. Microscopic observations showed etching effects on the surfaces of the metal reflectors after cleaning. In 2021, Fraxedas et al. [5] utilized low-pressure inductively coupled plasma for in situ cleaning of optical components in a synchrotron light source, using oxygen and argon as gas sources, and characterized surface element changes through in situ X-ray photoelectron spectroscopy. The interactions between reactive particles in the plasma and material surfaces that induce cleaning, etching, and modification processes occur at the nanosecond time scale and involve atomic-level interactions. Due to the lack of in situ characterization techniques for plasma physical processes, there are limitations in understanding the complex interactions between plasma and material surfaces. Therefore, Srinivasan et al. [22] employed reactive molecular dynamics (MD) methods to investigate the etching of diamond by high-temperature oxygen plasma, revealing the layer-by-layer etching mechanism of diamond. Xu et al. [23] from Tohoku University in Japan investigated the plasma etching mechanism of diamond using the ReaxFF force field. Simulation results indicated that irradiation by oxygen plasma disrupts the carbon bonds in diamond, eventually causing small molecular groups to desorb from the diamond surface. Xu et al. [23] utilized the ReaxFF force field to investigate the etching of diamond by oxygen and hydrogen plasmas, emphasizing the chemical reactions between the plasma and diamond (primarily composed of carbon). In our preliminary studies on the chemical reactions between various types of plasma and organic contaminants (primarily composed of carbon, hydrogen, and oxygen elements), we also employed the ReaxFF force field for description [24,25]. Molecular dynamics simulations provide a novel approach for studying the physical interactions between plasma and material surfaces.

This study focuses on fused silica, a typical optical component material, to mitigate the damage caused to optical component surfaces by excessive cleaning after removing organic contaminants by plasma. Molecular dynamics simulations uncover the patterns and mechanisms of oxygen plasma-induced damage on fused silica surfaces. Through simulation techniques, the research explores the evolution of damage to fused silica substrates at the atomic level, elucidating the impact of plasma energy, plasma flux, and ambient temperature on the damage process. By identifying critical characteristic parameters for plasma cleaning and fused silica surface damage, the study aims to elucidate the microscale damage mechanisms during plasma cleaning and ultimately achieve high-quality, non-destructive cleaning of optical component surfaces.

## 2. Results Analysis

### 2.1. Microscopic Process of Surface Damage to Fused Silica by Plasma Cleaning

**Figure 1:**
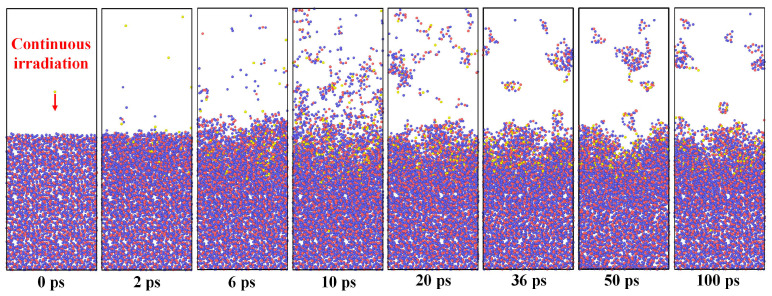
Reactive oxygen plasma and fused silica interaction process. The blue, red and yellow spheres represent the silicon and oxygen atoms in the silica coating and oxygen plasma, respectively.

Figure 1 illustrates the evolution of surface damage to fused silica caused by physical bombardment from oxygen plasma, with the kinetic energy of oxygen plasma set at 74 eV. Within the initial 10 ps of plasma deposition, as the plasma bombards persistently, the fused silica surface is gradually removed, silicon–oxygen bonds are disrupted, and silicon–oxygen atoms are sputtered from the surface. The fused silica sputtered by plasma bombardment is primarily distributed in atomic form within the model system. As the oxygen plasma bombardment ceases, the silicon–oxygen atoms sputtered from the fused silica surface gradually aggregate into small molecular clusters, dispersing in the vacuum environment. The damaged fused silica surface morphology remains unchanged after the reactive ion bombardment stops, with the surface damage region accumulating some oxygen plasma. Hence, focusing on the surface morphology of fused silica during plasma irradiation and the changes in atomic quantity is crucial.

**Figure 2:**
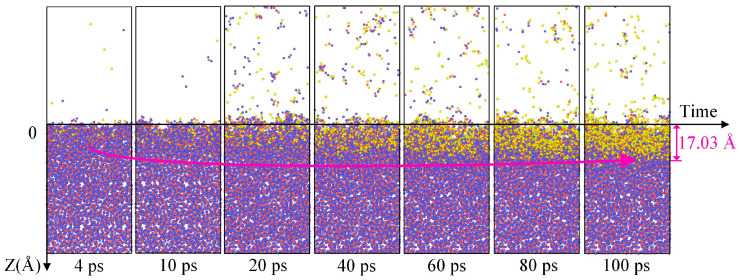
Continuous irradiation of reactive oxygen plasma for 100 ps. The blue, red and yellow spheres represent the silicon and oxygen atoms in the silica coating and oxygen plasma, respectively.

The simulation results from Figure 1 reveal a significant impact of the duration of plasma irradiation on induced damage to the fused silica surface. Consequently, the continuous plasma irradiation time in the simulation model was extended to 100 ps to analyze the effect of irradiation time on the damage to the fused silica substrate under the same energy and flux conditions. Although the simulation time in both Figure 1 and Figure 2 lasted for 100 ps, in Figure 1, the oxygen plasma irradiation lasted only 10 ps, with the remaining 90 ps representing free atomic motion within the model. In Figure 2, the oxygen plasma irradiation persisted for the full 100 ps. As depicted in Figure 2, the damage depth of the fused silica substrate gradually approaches a plateau with the continued irradiation of neutral oxygen atoms, reaching a damage depth of 17.03 Å at 100 ps. Following prolonged irradiation, neutral oxygen atoms are injected into the fused silica substrate, with the injection depth closely mirroring the damage depth. With increasing plasma irradiation time, the quantity of silicon–oxygen atoms sputtered from the substrate surface remains relatively stable, showing no significant increase. During short-duration oxygen plasma irradiation, the plasma transfers kinetic energy to the silicon and oxygen atoms within the underlying fused quartz, leading to the breaking of silicon–oxygen chemical bonds and the generation of atomic sputtering. With an increase in the duration of oxygen plasma irradiation, more oxygen plasma is injected into the silicon dioxide substrate, where subsequent oxygen ions bombard the already deposited oxygen ions within the substrate, forming a protective layer on the silicon–oxygen chemical bonds. Therefore, under the same plasma energy and flux, the sustained irradiation of neutral oxygen atoms on the fused silica substrate results in the damage depth gradually reaching a stable value as time progresses. Therefore, for more efficient exploration of surface damage on fused quartz during plasma cleaning processes in subsequent simulation studies, we adopted a setting involving 10 ps of continuous oxygen plasma irradiation followed by a relaxation period of 90 ps.

### 2.2. Interaction Process Between Plasma and Fused Silica Surfaces

**Figure 3:**
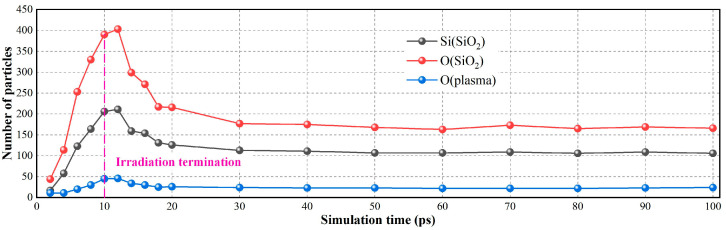
The interaction characteristics between oxygen plasma and fused silica.

Figure 3 presents a statistical analysis of the variation in the quantities of neutral oxygen atoms and silicon–oxygen atoms in fused silica surfaces during the 100 ps of plasma irradiation. Here, the statistics of particle numbers refer to atoms sputtered from the silicon dioxide substrate surface, including both silicon dioxide and oxygen plasmas. Within the initial 10 ps of continuous plasma bombardment, there is a linear increase in the quantity of neutral oxygen atoms, consistent with the release pattern of the plasma as detailed in the simulation.

As the plasma continues its bombardment, the quantity of silicon–oxygen atoms sputtered from the fused silica substrate surface exhibits a linear relationship. Moreover, the ratio of oxygen atoms to silicon atoms is 2:1, consistent with the atomic composition ratio of fused silica. After simulating 10 ps, the release of neutral oxygen atoms in the simulation model ceases. During the simulation process, a tiny fraction of atoms may exit the model and not return. These atoms are promptly removed to ensure the simulation’s operation. Consequently, during the simulation from 10 ps to 100 ps, the quantity of reactive oxygen atoms gradually decreases. The amount of silicon–oxygen atoms sputtered from the fused silica substrate surface decreases gradually, stabilizing numerically at 100 ps. However, the ratio of oxygen and silicon atoms sputtered from the substrate surface is 1.5:1, indicating that silicon atoms are more prone to breaking free from the constraints of the fused silica molecular structure.

**Figure 4:**
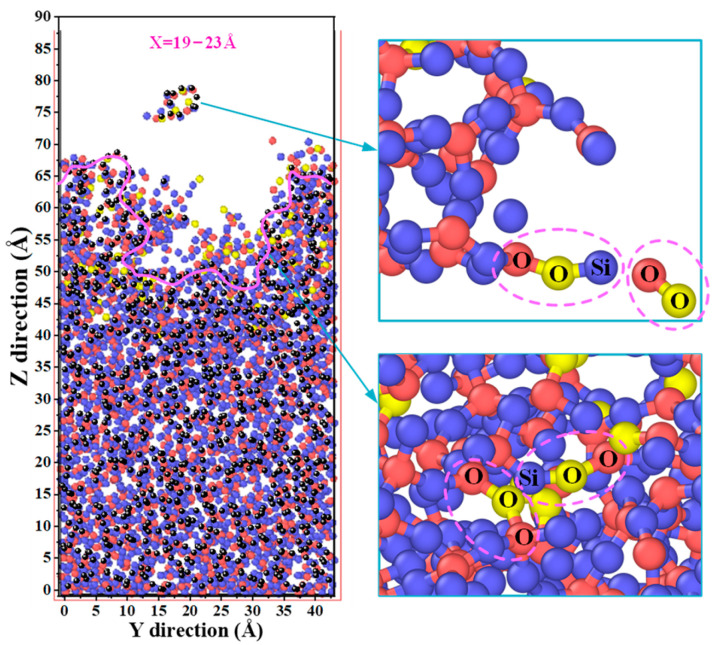
Cross-sectional morphology of the fused silica model after simulating 100 ps. The black atoms represent the silicon and oxygen atoms in the cross-section of the silicon dioxide substrate. The damaged morphology was connected with a pink solid line. The blue, red and yellow spheres represent the silicon and oxygen atoms in the silica coating and oxygen plasma, respectively.

After 10 ps of reactive oxygen ion irradiation of the fused silica substrate, the interaction system between the plasma and fused silica reaches a stable state at 100 ps. The cross-sectional view of the simulation model in Figure 4 reveals the formation of a pit defect in the amorphous fused silica substrate, with the surface porosity of fused silica increasing gradually with the duration of reactive oxygen ion irradiation. Concurrently, new Si-O bonds and O-O bonds form between the neutral oxygen atoms and the fused silica substrate, as well as the sputtered silicon–oxygen atoms.

### 2.3. Influence of Plasma Energy on Surface Damage to Fused Silica

**Figure 5:**
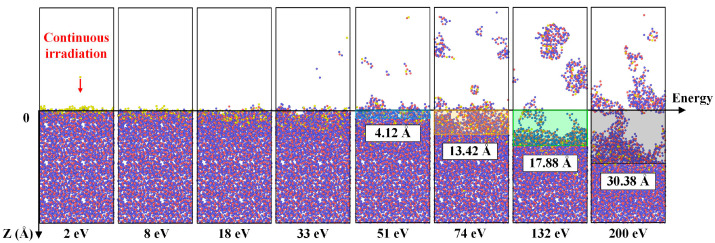
Influence of plasma incident energy on damage to the fused silica surface. Each snapshot was obtained after subjecting the system to continuous oxygen plasma bombardment for 10 ps, followed by a relaxation period of 90 ps. The blue, red and yellow spheres represent the silicon and oxygen atoms in the silica coating and oxygen plasma, respectively.

Figure 5 illustrates the impact of plasma energy on the substrate damage of fused silica. The damage caused by neutral oxygen atoms to the fused silica surface does not follow a linear relationship, while rather significant damage occurs only when the plasma energy exceeds 51 eV. Oxygen ions impinge perpendicularly onto the material surface, leading to ion implantation when the plasma energy ranges between 2 eV and 33 eV, with the depth of ion implantation increasing with the energy of the neutral oxygen atoms. When the plasma energy exceeds 33 eV, substantial damage occurs on the fused silica surface, resulting in sputtering of surface silicon–oxygen atoms.

At lower plasma incident energies, the fused silica surface experiences sputtering of small molecular clusters composed of silicon–oxygen. As the plasma incident energy gradually increases to 200 eV, the fused silica surface undergoes block-like sputtering of atoms, leading to thinning of the fused silica substrate. Furthermore, the increase in plasma incident energy does not exhibit a linear relationship with the depth of damage on the silica surface; rather, as the plasma energy increases, the depth of damage on the silica surface sharply rises.

**Figure 6:**
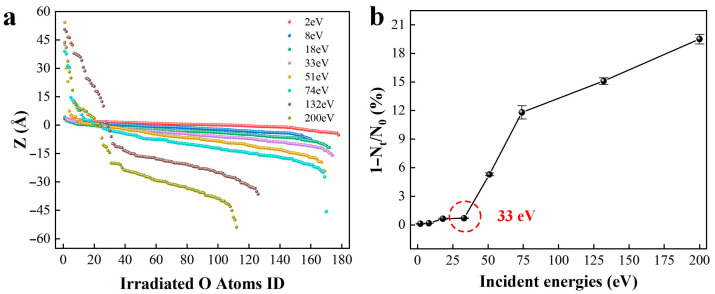
Data statistics of plasma incident energy damage to fused silica substrate: (**a**) the influence of incident energy on the deposition depth of oxygen free radicals; The statistical analysis of oxygen atom deposition depth under each plasma energy impact considered the last 5 ps and was subsequently averaged. (**b**) The influence of incident energy on the atomic loss rate.

Figure 6a presents a statistical analysis of the variation in the deposition depth of neutral oxygen atoms on the fused silica surface with increasing incident energy. The plasma deposits on the fused silica surface at lower ion incident energies without sputtering. As the incident energy of neutral oxygen atoms increases, the depth of ion deposition on the fused silica surface gradually increases. When the incident ion energy exceeds 33 eV, the sputtering of silicon–oxygen atoms on the fused silica surface leads to a greater ease of penetration of neutral oxygen atoms into the substrate material. Particularly, when the incident energy reaches 200 eV, neutral oxygen atoms deposit into the fused silica and sputter out a significant amount of oxygen atoms from the surface.

In the process of plasma bombardment, the thinning rate of fused silica is quantified through the “loss rate of silicon-oxygen atoms in the fused silica structure” defined as 1 − *N_t_*/*N*_0_, where *N_t_* and *N*_0_ represent the number of atoms lost in the fused silica substrate at times *t* and 0 ps, as shown in Figure 6b [23]. By comparing the effects of different oxygen free radical incident energies on the loss rate of fused silica atoms in the substrate, it is observed that when the oxygen free radical incident energy exceeds 33 eV, there is a sudden change in the fused silica surface atoms, with significant sputtering of silicon–oxygen atoms on the surface. With the continuous increase in atomic oxygen incident energy, the rate of fused silica thinning is positively correlated with the particle incident energy.

### 2.4. Influence of Plasma Flux and Ambient Temperature on Surface Damage to Fused Silica

**Figure 7:**
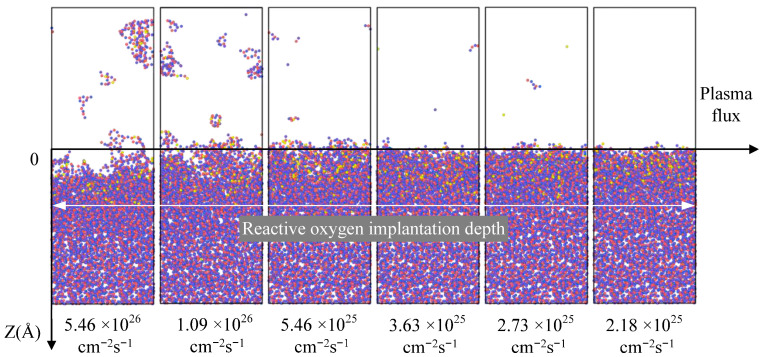
Influence of plasma flux on damage to the fused silica surface. Each snapshot was obtained after subjecting the system to continuous oxygen plasma bombardment for 10 ps, followed by a relaxation period of 90 ps. The blue, red and yellow spheres represent the silicon and oxygen atoms in the silica coating and oxygen plasma, respectively.

In the plasma cleaning process, besides the significant damage caused to the fused silica surface by plasma energy, the influence of plasma flux on the substrate damage of optical components also needs to be considered. Figure 7 depicts the damage to fused silica substrates under different plasma densities. The calculation of plasma flux can be understood as the interval time for individual particles to enter a unit area. Thus, a particle incident energy was set as 74 eV and a plasma flux was set as 1.09 × 10^26^ cm^−2^ s^−1^ in this section.

According to the simulation results shown in Figure 5, it is evident that a critical damage threshold occurs when the plasma incident energy surpasses the binding energy of the fused silica substrate. As the plasma flux increases, the degree of surface damage to the fused silica substrate gradually intensifies without exhibiting significant critical damage features. However, with an increase in plasma flux, the silicon dioxide substrate does not exhibit such sudden damage. Following the conclusion of oxygen plasma irradiation, the penetration depth of the oxygen plasma remains relatively consistent. This implies that in the plasma cleaning procedure, the primary influencing factor determining the depth of plasma injection, and thus the depth of surface damage to optical components, is the plasma incident energy, while plasma flux exacerbates the rate of surface damage to fused silica during the plasma cleaning process. The surface damage to optical elements caused by physical effects during plasma cleaning is jointly determined by both the plasma incident energy and flux.

**Figure 8:**
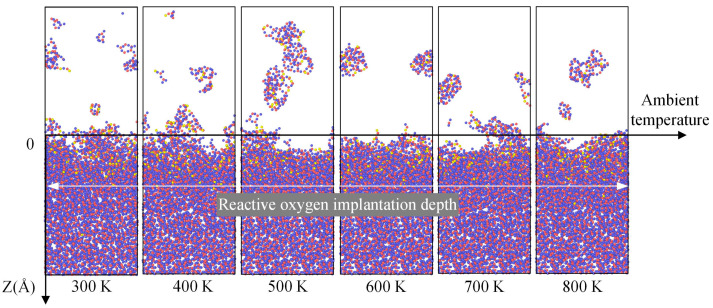
Influence of ambient temperature on silica surface damage during plasma cleaning. Each snapshot was obtained after subjecting the system to continuous oxygen plasma bombardment for 10 ps, followed by a relaxation period of 90 ps. The blue, red and yellow spheres represent the silicon and oxygen atoms in the silica coating and oxygen plasma, respectively.

The actual heating of the overall system accelerates the rate of organic contaminant removal. However, whether the heating environment affects surface damage to fused quartz during the cleaning process requires further discussion through simulation. Our investigation into the three parameters of plasma energy, flux, and ambient temperature involved studying each factor individually. For the study on the effect of ambient temperature on the damage of fused quartz during the plasma cleaning process, the energy of the oxygen plasma was set at 74 eV, with a flux of 1.09 × 10^26^ cm^−2^ s^−1^. Figure 8 illustrates the impact of ambient temperature on surface damage to fused silica during the plasma cleaning process. From the figure, it can be observed that despite the increase in ambient temperature from 300 K to 800 K, which intensifies the condensation of sputtered silicon–oxygen atoms, it has no significant effect on the thinning depth of the fused silica substrate or the depth of ion injection. This is because while the increase in ambient temperature aids in boosting the energy of neutral oxygen atoms to some extent, the effect is quite limited. Therefore, within a specific range, raising the ambient temperature during the plasma cleaning process does not heighten the risk of damage to the substrate of optical elements. However, it does accelerate the rate of aggregation and precipitation of sputtered silicon–oxygen atoms on the surface of fused silica.

## 3. Simulation Model

The bulk fused silica model in the simulation was created based on the actual manufacturing process of fused silica optical components. Initially, we heated α-quartz to 4000 K for 100 ps, followed by cooling the molten silica in an NVT (N—particle number, V—volume, T—temperature) system from 4000 K to 300 K at a rate of 25 K/ps. Subsequently, the entire system underwent a 100 ps relaxation in an NPT (N—particle number, P—pressure, T—temperature) system at a temperature of 300 K and a pressure of 1 atm, resulting in the final fused silica bulk model with a density of approximately 2.2 g/cm^3^. This method of creating fused silica is widely utilized in the molecular dynamics simulations [26,27,28].

**Figure 9:**
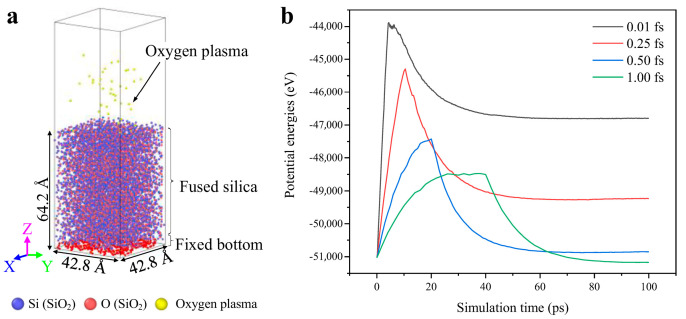
(**a**) Simulation model of oxygen plasma physical bombardment of the fused silica and (**b**) the variation in system potential energy as simulation time increases under various time steps.

A microscopic interaction model has been constructed, utilizing reactive oxygen species in air plasma as an example. The model delineates the interactions between plasma particles and the fused silica substrate, as illustrated in Figure 9a. Molecular dynamics simulations were employed to investigate the physical processes of continuous bombardment of fused silica surfaces by oxygen plasma. During the plasma cleaning process, contaminant removal primarily involves chemical reactions supplemented by physical bombardment. Theoretical and experimental analysis [3] suggests that the damage caused by oxygen plasma to fused quartz surfaces is predominantly due to physical bombardment. Hence, we utilize the Tersoff force field, which effectively describes the removal of surface atoms and changes in surface structure. The Tersoff force field, a classical force field, utilizes parameters derived from first-principles calculations to describe the interactions between atomic silicon and oxygen radicals [29]. The Tersoff force field has been successfully applied in various studies of silicon-based materials, including fused quartz, demonstrating exemplary performance in depicting the breaking and formation of silicon–oxygen bonds [30]. Moreover, compared to the ReaxFF force field, the Tersoff force field offers higher computational efficiency and is suitable for modeling the complex atomic interactions involved in plasma cleaning processes. It is particularly important to note that the Tersoff force field does not explicitly state that it can describe the interaction between oxygen atoms and Si-O. However, there are very few reports of this force field in the existing literature. Therefore, in this study, the parameters of high-energy oxygen atoms are based on those of oxygen atoms in the Tersoff force field in order to simplify the interaction relationship of this part. In the subsequent first-principles research, the parameters of high-energy oxygen atoms need to be further corrected. It is well known that oxygen, once ionized, contains various highly reactive species, including O^+^, O^−^, O^2−^, O, O_3_ (ionized ozone), excited O_2_, and electrons. However, due to the Auger neutralization effect, the plasma becomes neutral when it approaches the silicon dioxide substrate [31,32]. While we aimed to study all species and interactions between the substrate fused quartz, in classical MD, the Tersoff potential function can only simulate interactions of uncharged O atoms with silicon and oxygen atoms in the fused quartz. Considering that uncharged O atoms are the primary component of the oxygen plasma interacting with fused quartz, our MD simulations only consider uncharged O atoms to represent the interactions of the oxygen plasma with fused quartz.

All atomic entities involved in the simulation were placed within a box of dimensions 42.8 × 42.8 × 124.2 Å^3^, with the height of the amorphous silica being 64.2 Å, as shown in Figure 9a. The bottom layer of the substrate was anchored with a thickness of 3 Å to prevent basal movement during the simulation. A vacuum layer of 60 Å was added above the silica substrate, followed by the introduction of oxygen plasma. The simulation box was subjected to periodic boundary conditions in the X and Y directions to create an infinite simulation system. In contrast, the Z direction had fixed reflective boundaries to prevent the escape of neutral oxygen atoms. The entire simulation was conducted for 100 ps under the NVT ensemble. To demonstrate that the chosen timestep of 0.25 fs provides an acceptable balance between accuracy and efficiency, we performed an additional test. We ran three independent NVT trajectories (T = 300 K, 100 ps each) using timesteps of 0.10 fs, 0.25 fs, 0.50 fs, and 1.00 fs. The potential-energy traces are shown in the new Figure 9b. In a 100 ps simulation process, only the initial 10 ps constitute the period of plasma bombardment. Therefore, the corresponding significant decrease in system potential energy during the simulation process should occur after 10 ps. From Figure 9b, it is evident that with time steps of 0.01 fs and 0.25 fs, the system potential energy rapidly rises within the first 10 ps, followed by a swift decline, aligning with the settings in the actual simulation process. As the time step increases to 0.50 fs and 1.00 fs, the system potential energy shows varying degrees of increase after 10 ps before trending downward. Although the trends are consistent, the computational accuracy is evidently inferior with time steps of 0.50 fs and 1.00 fs. Considering both computational accuracy and efficiency, a time step of 0.25 fs is selected for the simulation model. After initial conjugate-gradient energy minimization, the system was equilibrated for 0.05 ps under an NVT thermostat at 300 K to approximate working conditions. After energy minimization, the system was equilibrated in the NVT ensemble at 300 K for 0.05 ps, at which point the temperature and potential energy fluctuations had converged within 2% of their long-time averages, ensuring the starting configuration reflects typical working conditions. During deposition, we switch to an open-system protocol combining “fix deposit” (adds O atoms) and “fix temp/berendsen” (thermostat only). During the simulation process, only oxygen was supplied to the system, and the oxygen species left the system in an uncoordinated manner, which was an uncontrolled state. Therefore, the NVT state was maintained during the intervals when oxygen was supplied to the system. Additionally, to simulate heat exchange between the system and the external environment, the Berendsen method was employed during the simulation process to regulate temperature fluctuations within the model. Oxygen plasma in the simulation model was deposited, replicating the physical process of continuous particle bombardment on the surface during cleaning. All simulations in this chapter were executed using the LAMMPS (64-bit 28Mar2023) software [33], and post-processing of simulation data was performed using the OVITO (Basic) software [34].

Key parameters influencing the quality of plasma cleaning include plasma energy and flux, as well as variations in environmental temperature. Therefore, in the simulation process, the study of plasma energy is achieved by altering the incident velocity of oxygen plasma, as detailed in Equation (1). Research on plasma flux is conducted by varying the quantity of oxygen plasma incident on the fused quartz substrate per unit time. Investigation into environmental temperature is accomplished by adjusting the initial temperature of the entire simulation model. It is important to note that all oxygen plasma is incident perpendicular to the surface of the fused quartz substrate, randomly released within the range of 95–105 Å directly above the entire fused quartz substrate. Consequently, the area of fused quartz irradiated by oxygen plasma is consistent across all simulations. Considering the microscopic interactions between oxygen plasma and fused quartz in molecular dynamics simulations, we have set a broad range for the selection of oxygen plasma parameters to observe significant patterns of interaction on the picosecond time scale. This approach is widely utilized in other molecular dynamics simulation studies as well [22,23,24,25].

During the simulation, a reactive oxygen atom is inserted every 0.05 ps, with a flux of 1.09 × 10^26^ cm^−2^ s^−1^, totaling 200 reactive oxygen atoms released. Therefore, within the initial 10 ps of the simulation, all 200 reactive oxygen atoms have been released. The simulation continues for the subsequent 90 ps to allow the neutral oxygen atoms deposited on the silica surface to reach a stable equilibrium state. All oxygen plasma particles involved in interactions remain electrically neutral. The characteristics of the plasma are primarily determined by two parameters: plasma energy and plasma flux. The formula for calculating the kinetic energy of particles is shown in Equation (1). In the simulation, the incident velocities of oxygen ions are 50 Å/ps, 100 Å/ps, 150 Å/ps, 200 Å/ps, 250 Å/ps, 300 Å/ps, 400 Å/ps, and 500 Å/ps, corresponding to plasma energies of 2 eV, 8 eV, 18 eV, 33 eV, 51 eV, 74 eV, 132 eV, and 200 eV, respectively.

(1) $$E=\frac{1}{2}mv^{2}$$

where *E* stands for the ion’s kinetic energy, measured in electron volts (eV). The symbol *v* indicates the velocity of the ion, expressed in angstroms per picosecond (Å/ps). Meanwhile, *m* represents the mass of the ion, given in grams.

## 4. Conclusions

This study investigated the induced surface damage characteristics of optics by plasma cleaning to apply plasma technology for in situ cleaning of large-aperture optics in large-scale precision optics facilities. By employing molecular dynamics to explore the microscopic processes of plasma-induced surface damage on optics, insights into the surface damage mechanisms of optics and the thinning patterns of coatings during plasma cleaning are obtained. Molecular simulations reveal the damage mechanisms of reactive groups on the surface of optics during plasma cleaning. Energetic particle bombardment during plasma cleaning disrupts the chemical bonds of the fused silica substrate, sputtering silicon–oxygen atoms and gradually removing surface atoms of the fused silica layer by layer. Silicon atoms sputtered from the fused silica substrate surface are more easily detached from the molecular structure, and the number of sputtered atoms exhibits a linear relationship with irradiation time. With increasing irradiation time of neutral oxygen atoms, pit defects form in the middle of the fused silica surface. The characteristic patterns of interface damage on optics during plasma cleaning have been obtained. Significant damage features on the fused silica substrate only occur when the energy of neutral oxygen atoms exceeds 33 eV, accompanied by plasma injection into the coating layer. With the gradual increase in plasma incident energy, block-like molecular sputtering occurs on the fused silica surface, leading to layer-by-layer thinning. Plasma energy plays a decisive role in surface damage to fused silica during plasma cleaning. In the future processes of plasma cleaning fused quartz optical elements, to enhance cleaning efficiency while avoiding surface damage caused by cleaning, it may be considered to control the plasma energy within 33 eV while simultaneously increasing the plasma flux and environmental temperature.

## Data Availability

The datasets generated and/or analysed during the current study are available upon reasonable request. Researchers interested in accessing the data can contact the corresponding authors.
